# Differential response to donepezil in MRI subtypes of mild cognitive impairment

**DOI:** 10.1186/s13195-023-01253-2

**Published:** 2023-06-23

**Authors:** Patricia Diaz-Galvan, Giulia Lorenzon, Rosaleena Mohanty, Gustav Mårtensson, Enrica Cavedo, Simone Lista, Andrea Vergallo, Kejal Kantarci, Harald Hampel, Bruno Dubois, Michel J. Grothe, Daniel Ferreira, Eric Westman

**Affiliations:** 1 Department of Radiology, Mayo Clinic, Rochester, MN USA; 2 Division of Clinical Geriatrics, Center for Alzheimer Research, Department of Neurobiology, Care Sciences and Society, Karolinska Institutet, Huddinge, Sweden; 3 Alzheimer Precision Medicine (APM), Sorbonne University, AP-HP, Pitié-Salpêtrière Hospital, Boulevard de L’hôpital, Paris, France; 4 Unidad de Trastornos del Movimiento, Servicio de Neurología y Neurofisiología Clínica, Instituto de Biomedicina de Sevilla, Hospital Universitario Virgen del Rocío, CSIC, Sevilla, Spain; 5 Wallenberg Center for Molecular and Translational Medicine, Department of Psychiatry and Neurochemistry, University of Gothenburg, Gothenburg, Sweden; 6 Department of Neuroimaging, Centre for Neuroimaging Sciences, Institute of Psychiatry, Psychology, and Neuroscience, King’s College London, London, UK

**Keywords:** Randomized controlled trial, Donepezil, Mild cognitive impairment, Heterogeneity, Subtypes, Alzheimer’s disease, Precision medicine

## Abstract

**Background:**

Donepezil is an approved therapy for the treatment of Alzheimer’s disease (AD). Results across clinical trials have been inconsistent, which may be explained by design-methodological issues, the pathophysiological heterogeneity of AD, and diversity of included study participants. We investigated whether response to donepezil differs in mild cognitive impaired (MCI) individuals demonstrating different magnetic resonance imaging (MRI) subtypes.

**Methods:**

From the Hippocampus Study double-blind, randomized clinical trial, we included 173 MCI individuals (donepezil = 83; placebo = 90) with structural MRI data, at baseline and at clinical follow-up assessments (6–12-month). Efficacy outcomes were the annualized percentage change (APC) in hippocampal, ventricular, and total grey matter volumes, as well as in the AD cortical thickness signature. Participants were classified into MRI subtypes as typical AD, limbic-predominant, hippocampal-sparing, or minimal atrophy at baseline. We primarily applied a subtyping approach based on continuous scale of two subtyping dimensions. We also used the conventional categorical subtyping approach for comparison.

**Results:**

Donepezil-treated MCI individuals showed slower atrophy rates compared to the placebo group, but only if they belonged to the minimal atrophy or hippocampal-sparing subtypes. Importantly, only the continuous subtyping approach, but not the conventional categorical approach, captured this differential response.

**Conclusions:**

Our data suggest that individuals with MCI, with hippocampal-sparing or minimal atrophy subtype, may have improved benefit from donepezil, as compared with MCI individuals with typical or limbic-predominant patterns of atrophy. The newly proposed continuous subtyping approach may have advantages compared to the conventional categorical approach. Future research is warranted to demonstrate the potential of subtype stratification for disease prognosis and response to treatment.

**Trial registration:**

ClinicalTrial.gov NCT00403520. Submission Date: November 21, 2006.

**Supplementary Information:**

The online version contains supplementary material available at 10.1186/s13195-023-01253-2.

## Introduction

Donepezil is an acetylcholinesterase inhibitor (AChE-I) approved and standard-of-care for the symptomatic treatment of patients with Alzheimer’s disease (AD) dementia. Diverse clinical trials have demonstrated that donepezil has moderate effects on cognitive performance at different clinical symptomatic stages of AD [1–4]. The effect of AChE-Is on cognition is persistent over time and is associated with delayed institutionalization and reduced mortality in dementia [5–7]. These findings have been discussed mainly under the cholinergic deficit hypothesis. According to this hypothesis, symptoms of dementia in AD patients are explained by the lack of acetylcholine neurotransmitter because of reduced synthesis and release of acetylcholine, which leads to the death of neuronal cells. Donepezil increases the availability of acetylcholine by inhibiting the acetylcholinesterase, an enzyme responsible for the acetylcholine catabolism in the synaptic space. Therefore, increasing acetylcholine levels may improve cognitive symptoms in AD [8]. Experimental and clinical studies have also shown that donepezil acts at a molecular, cellular, brain structural, and functional network level, with a hypothetical biological effect on AD pathophysiological changes [9–14]. Studies also suggest better long-term cognitive and functional outcomes if donepezil treatment starts in early clinical stages of AD [15].

Notwithstanding this cross-disciplinary evidence, clinical trials of donepezil in prodromal stages of AD have provided inconsistent data so far [15]. These discrepancies may be explained by trial design insufficiencies and/or poor uniformity in eligibility criteria, with consequent inclusion of heterogeneous AD populations across trials, alongside a lack of biomarker-based outcome and endpoint assessment. Hence, recognizing disease heterogeneity (for example, the existence of biological subtypes of AD) is a critical step forward, towards the development of precision medicine and will be important for optimized future healthcare and clinical trial designs [16].

**Fig. 1:**
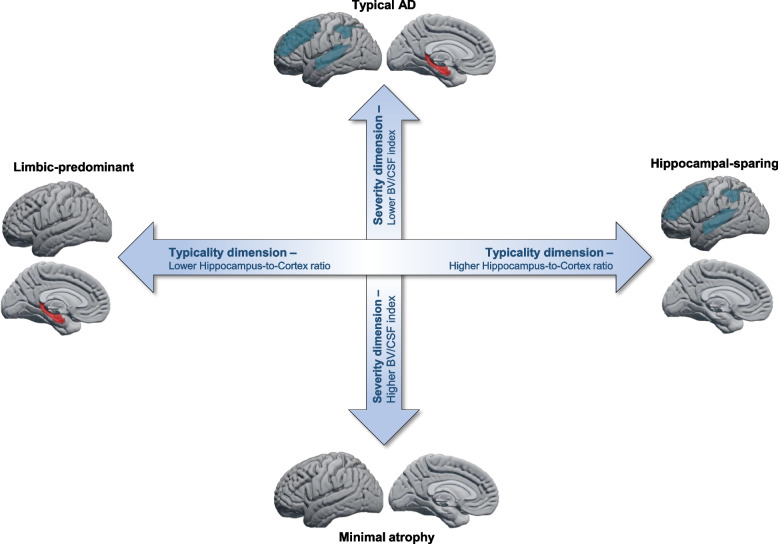
Conceptual framework of AD subtyping dimensions. In this study, we selected a global brain atrophy index (BV/CSF index; [29]) as a proxy for the severity dimension, and the ratio between the volume of the hippocampus to the volume of the cortex as a proxy for the typicality dimension [18, 19]. The combination of severity and typicality dimensions indicates four distinct MRI patterns: typical AD, limbic-predominant, hippocampal-sparing, and minimal atrophy. Adapted from Ferreira et al. [17]

The study of biological subtypes in AD has opened a window to unravel the disease heterogeneity [17]. Pathological and neuroimaging (magnetic resonance imaging (MRI) and positron emission tomography (PET)) studies have consistently identified three subtypes of AD based on the regional distribution of tau neurofibrillary tangles (NFT) or pattern of brain atrophy: *typical AD*, *limbic-predominant AD*, and *hippocampal-sparing* AD [18–20]. Several studies also identified a fourth subtype characterized by *minimal tau load or atrophy* [21–25]. Classification of AD patients into these biological subtypes has yielded promising results for a more accurate prediction of response outcomes in clinical trials [26]. However, a common caveat in previous studies is the use of arbitrary cut points on AD biomarkers to define biological subtypes in AD [24]. The use of cut points for interpretation of AD biomarkers might lead to misclassification of individuals when using categorical groups [21, 27]. Using arbitrary cut points and categorical groups could be avoided by using the current conceptual framework, which describes disease heterogeneity in two dimensions that are continuous in nature: typicality and severity (see Fig. 1) [17]. The typicality dimension ranges from limbic-predominant- to hippocampal-sparing-like subtypes, while the severity dimension goes from minimal atrophy/tau- to typical AD-like subtypes. There is data supporting the usefulness of continuous subtyping over the conventional categorical subtyping. For example, a recent study showed that continuous subtyping better captured the association between tau-positron emission tomography (PET) patterns and brain atrophy than the conventional categorical subtyping [28].

The main objective of this study is to investigate the response to donepezil in different MRI subtypes of individuals with amnestic mild cognitive impairment (MCI), which is considered the prodromal stage of AD dementia [30]. We subtyped the individuals using the new continuous scale approach, but we also report the results from the conventional categorical subtyping approach for comparison. We investigated response to donepezil on primary and secondary outcomes in our subtypes. The primary outcome was the rate of atrophy over 1 year of donepezil treatment. The secondary outcome was cognitive decline, also over 1 year of treatment. We hypothesized that (i) Using continuous scales for defining MRI severity and typicality dimensions would better capture the effects of donepezil treatment among MRI subtypes than the conventional categorical approach; (ii) Based on previous studies suggesting that cholinergic treatment has a greater effect in individuals that have less atrophy in medial temporal lobe structures [31], we anticipated that MCI individuals towards minimal atrophy- or hippocampal-sparing-like MRI patterns would show a more positive effect of donepezil treatment than MCI individuals towards limbic-predominant- and typical AD-like MRI patterns.

## Methods

### Study population

**Fig. 2:**
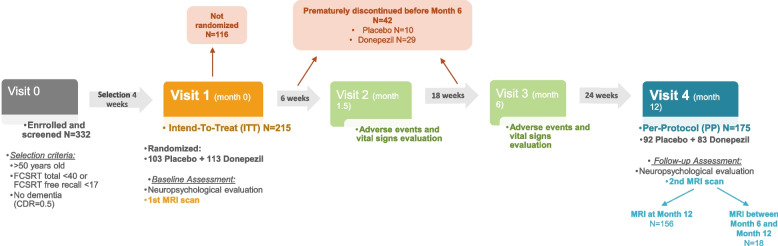
Design and protocol of the Hippocampus Study Clinical Trial (www.clinialTrial.gov; identifier: NCT00403520). The Hippocampus Study Clinical Trial extended for up to 18 months, consisting of a 4-week selection period (visit 0), a 12-month randomized double-blind treatment window (visits 1–4), and an open label extension period of 6 more months (visits 4–5). At the treatment window, individuals were randomly assigned to either the active treatment group or the placebo control group. The treatment group received 1 capsule of 5-mg donepezil daily from week 0 to 6, then 2 capsules of 5-mg donepezil (i.e. 10 mg) until month 12; the placebo group received 1 placebo capsule daily until week 6, and then two placebo capsules until month 12

Individuals with MCI were collected from the “Hippocampus Study Clinical Trial”. The “Hippocampus Study” is a multicentre double-blind, randomized, placebo-controlled trial (www.clinialTrial.gov; identifier: NCT00403520) that started in November 2006 and concluded in August 2010. The “Hippocampus Study” was conducted within a French network of Memory Resources and Research Centers (CMRR) that enhances 28 centres in France with neurologists, geriatricians, and neuropsychologists, as well as biological and neuroimaging resources. The design of the trial is summarized in Fig. 2. Further information on the design and protocol of the “Hippocampus Study Clinical Trial” is detailed in previous publications [12–14].

The per-protocol population (*n* = 173, Dubois et al. 2015) was selected for our current study. The per-protocol population has MRI scans and neuropsychological evaluations at baseline (visit 1) and at 12-month follow-up (visit 4). All individuals met the inclusion criteria at enrollment phase (visit 0): (1) older than 50 years of age; (2) have a progressive hippocampal amnestic syndrome defined by Free Recall score ≤ 17 or Total Recall score < 40 on the Free and Cued Selective Reminding Test (FCSRT) [32]; (3) no dementia, with a clinical dementia rating (CDR) ≤ 0.5; and 4) preserved global cognitive performance according to a score on Mini-Mental State Examination (MMSE) > 24 and preserved Instrumental Activities of Daily Living according to specific questionnaires (Dubois et al. 2015).

The Hippocampus Study Clinical Trial was approved by the institutional review board from each of the participating research centres and by the Ethic Committee of the Coordination Centre at La Pitié-Salpetriere Hospital, Paris, France (Reference Number: E2020-E033-41, Ethic Committee Approval: November 8, 2006). The study was conducted in accordance with the Declaration of Helsinki and all individuals provided written consent.

### MRI acquisition

An MRI was performed using 1.5 T or 3 T MRI scanners qualified by the central MRI analysis core at the Cogimage Team, *Institut du Cerveau et de la Moelle Épiniere*. A standardized protocol for image acquisition was used at each site as previously described [12]. For the current study, we selected 3D T1-weighted images with an isotropic voxel size of 1.3 mm for scans at 1.5 T and 1 mm for scans at 3 T. To minimize the scanner-related variability, each individual was evaluated with the same scanner at baseline (visit 1) and at 12-month follow-up (visit 4). After each acquisition, expert neuro-radiologists inspected the images for potential artifacts of movements, ringing, wrap around, and metal artifacts.

### MRI processing

T1-weighted images were pre-processed using FreeSurfer (version 6.0; http://freesurfer.net/) through the HiveDB database system [33] following standard processing steps [34]. Careful visual quality control was performed on both the original and processed data. Measures of volume and thickness were extracted for 34 cortical regions from both hemispheres, and measures of volume were extracted for 21 subcortical regions using the Desikan atlas [35]. We also obtained the estimated total intracranial volume (ICV) from FreeSurfer to adjust volume values by head size using the residual approach [36].

### Characterization of AD heterogeneity

#### Using continuous scales

We primarily investigated MRI heterogeneity in our cohort by quantifying the dimensions of *severity* and *typicality* of MRI patterns using continuous scales. *Severity* was proxied by the brain volume-to-cerebrospinal fluid volume (BV/CSF) index [29], and *typicality* was proxied by the hippocampus-to-cortex ratio [18, 19].

The BV/CSF index was calculated by dividing the total volume of the brain (BV) by the total volume of the cerebrospinal fluid (CSF) as described in a previous study [29]. The total BV was calculated by summing up the volume of the grey and white matter. Lower values of the BV/CSF index indicate more atrophy. Age-adjusted clinical cut-offs for the BV/CSF index have been published to determine the degree of severity as “No atrophy”, “Mild atrophy”, “Moderate atrophy”, and “Severe atrophy” [29]. In the current study, we applied the age-adjusted clinical cut-offs only for descriptive purposes. In the context of this study, lower values of the BV/CSF index indicate a more typical AD-like MRI pattern and higher values indicate a more minimal atrophy MRI pattern. The BV/CSF index has previously been used in an AD clinical trial of a nerve growth factor treatment targeting the cholinergic system [37].

The hippocampus-to-cortex ratio was calculated by dividing the averaged volume of the left and right hippocampus by the averaged volume of 3 cortical regions [18, 19]: (1) middle frontal gyri; (2) inferior parietal gyri; (3) and superior temporal gyri. Lower values indicate more atrophy of the hippocampus over the cortex (i.e. towards the limbic-predominant pattern [28]), while higher values denote more atrophy of the cortex over the hippocampus (i.e. towards the hippocampal-sparing pattern [23]).

#### Using the conventional categorical approach

Complementary, we also characterized MRI subtypes following the conventional categorical approach [22]. We combined visual rating scales of regional brain atrophy to assign participants into one of four categorical subtypes: *typical AD*, *limbic-predominant*, *hippocampal-sparing*, or *minimal atrophy.* To obtain the visual rating scale scores, we used AVRA (Automatic Visual Ratings of Atrophy v0.8 toolbox; https://github.com/gsmartensson/avra_public; [38]. AVRA is a deep learning model that computes rating estimations for three visual rating scales: the medial temporal atrophy scale (MTA; [39]), which assesses atrophy in the medial temporal lobe; the posterior atrophy scale (PA; [40]), which assesses atrophy in the posterior cortex; and the global cortical atrophy scale-frontal subscale (GCA-F; [41, 42]), which assesses atrophy in the frontal lobe. Scores were considered normal or abnormal using cut points described elsewhere [22]. Combinations of MTA, PA, and GCA-F scores defined the categorical subtypes. Briefly, for the MTA scale, a score of ≥ 1.5 was considered abnormal for age range of 45–74 years, a score ≥ 2 was considered abnormal for age range 75–84 years, and a score ≥ 2.5 was considered abnormal for age range 85–94 years. For PA and GCA-F, a score of ≥ 1 was considered abnormal irrespectively of the range of age. Using these cut points, the subtypes were defined as follows:

- *Typical AD* subtype: abnormal MTA together with abnormal PA and/or abnormal GCA-F.
- *Limbic-predominant* subtype: abnormal MTA with normal PA and normal GCA-F.
- *Hippocampal-sparing* subtype: abnormal PA and/or abnormal GCA-F, but normal MTA.
- *No-atrophy* subtype: normal scores in MTA, PA, and GCA-F.

### Donepezil efficacy measures

Based on the study protocol of the Hippocampus Study [12], the primary efficacy outcome of donepezil treatment was the annual percentage of change (APC) of the following MRI measures: ICV-adjusted volumes of the hippocampus, lateral ventricles, and total grey matter. To evaluate the impact of donepezil on AD-related cortical thinning, we calculated the AD signature cortical thickness including entorhinal, inferior temporal, middle temporal, and fusiform gyri thickness, as described in the literature [43].

APCs of the MRI efficacy measures were computed as follows:

$$\mathrm{APC}=\frac{\text{value at 12-month follow-up }-\;\mathrm{value}\;\mathrm{at}\;\mathrm{baseline}\;}{\mathrm{value}\;\mathrm{at}\;\mathrm{baseline}}\times\;\frac{365}{\mathrm{MRI}\;\mathrm{interval}}\times100$$

APC represents the rate of change in the MRI measures in 1 year, counting from baseline (visit 1) to 12-month follow-up (visit 4). APC ranges from − 100 to 100%. Negative APC values indicate greater loss of volume or cortical thickness (i.e. greater degeneration) over 1 year. Almost all the individuals underwent baseline and follow-up MRI assessments within 1 year (mean = 11.6 months). However, a small number (10%) underwent the MRI scan earlier, between 6 and 11 months after baseline. To minimize this variability in follow-up time, we calculated the APCs accounting for the number of days passed between the first MRI at baseline and the follow-up MRI scan (i.e. “MRI interval” in the formula).

The secondary efficacy measure was the percentage of change in cognitive outcomes. Clinical and cognitive measures were the ADAS-COG-MCI, MMSE, Isaacs’s verbal fluency and lexical fluency tests (15- and 60-item versions), TMT-Part A and B, and the Benton test, as in the original Hippocampus Study design [12]. These tests have been described in previous reports [12, 44]. The percentage of change (PC) was calculated as follows:

$$\mathrm{PC}=\frac{\text{value at 12-month follow-up }-\text{ value at baseline}}{\text{value at baseline}}\times 100$$

### Statistical analysis

Characteristics of placebo and donepezil-treated groups were described as means and standard deviations for continuous variables, and as counts and percentages for categorical variables. Differences in characteristics between placebo and donepezil-treated groups were evaluated using one-way ANOVA or Kruskal–Wallis for continuous variables, and the chi-square test for categorical variables.

We used multiple linear regression testing for interactions (backwards, with the best general lineal model—*bestglm*—method) to assess our main objective: to investigate whether the effect of donepezil treatment varies along the MRI subtyping dimensions, which are defined using continuous scales of typicality and severity. We fitted a regression model for each efficacy measure. The dependent variable in each regression model was the APC in each MRI measure or PC in each cognitive measure. The predictors were the treatment group (coded as a dummy variable; 0-placebo and 1-donepezil), the BV/CSF index as the proxy of the severity dimension, and the hippocampus-to-cortex ratio as the proxy of the typicality dimension. Age was included as co-variate in all models, as well as MRI field strength in those models where the dependent variable was the APC of MRI measures. Our effects of interest in the regression models were the interaction of subtyping dimensions (BV/CSF index or hippocampus-to-cortex ratio) with the treatment variable (placebo or donepezil) in the prediction of each efficacy measure.

Complementary, we also studied differences in the effect of donepezil treatment across MRI subtypes defined using the conventional categorical approach. Mixed ANOVAs were performed to assess the interaction between the categorical subtypes (between-subject factor, four levels: *typical AD, limbic-predominant AD, hippocampal-sparing AD*, or *minimal atrophy AD*) and the treatment variable (between-subject factor, two levels: donepezil and placebo groups) for each efficacy measure. Age was included as co-variate, as well as MRI strength field when dependent variable was the APC of the MRI measures. *P* values in all post hoc analyses were adjusted using the Benjamini–Hochberg correction for multiple comparisons.

All statistical analyses were performed using R 4.0.2, and results were deemed significant when *p* ≤ 0.05.

## Results

### Baseline characteristics

Baseline characteristics of the per-protocol population are displayed in Table 1. Donepezil and placebo groups did not differ in demographic characteristics (age, sex, and education), depressive symptomatology, cognitive performance, and MRI measures at baseline. The apolipoprotein E (*APOE*) genotype was available for 64 (37%) individuals (placebo, *n* = 36; donepezil, *n* = 28). Results showed no significant difference in the proportion of carriers of at least one *APOE* ɛ4 allele between donepezil and placebo groups.

**Table 1:** Baseline characteristics

	**Placebo** **(*n* = 90)**	**Donepezil** **(*n* = 83)**	***p* value**
Age,	73.7 (6.6)	73.9 (6.6)	0.595
Sex, female, *n* (%)	45 (53)	44 (51)	0.866
Education, *n* (%)			0.213
No education	0 (0)	1 (1)	-
Primary	7 (8)	6 (7)	-
Certification of primary	31 (37)	42 (47)	-
Secondary	23 (28)	13 (15)	-
Higher education	22 (27)	27 (30)	-
Duration of memory disorders	39.0 (28.6)	31.8 (24.7)	0.079
Hamilton Depression Rating Scale	3.2 (3.0)	2.8 (2.5)	0.807
*APOE* ε4 carriers, n (%) ^a^	16/28 (57)	16/36 (44)	0.450
Follow-up MRI (months)	11.5 (1.6)	11.7 (1.2)	0.746
MMSE	25.8 (2.6)	26.2 (2.1)	0.359
ADAS-COG-MCI	12.1 (4.2)	12.0 (4.3)	0.835
TMT-A (Time)	63.4 (31.1)	61.4 (26.9)	0.857
TMT-B (Time)	156.8 (65.5)	143.7 (58.1)	0.238
Benton test	6.9 (2.0)	7.1 (1.9)	0.439
Isaacs test (15 items)	24.7 (5.2)	25.0 (5.2)	0.692
Isaacs test (60 items)	49.8 (10.8)	50.0 (12.8)	0.818
Field strength 3 T, *n* (%)	22 (25)	23 (28)	0.785
Total hippocampal volume	6270.1 (904.8)	6275.3 (807.2)	0.851
Total grey matter volume	549,962.0 (29,424.3)	554,866.0 (32,668.1)	0.300
Lateral ventricular volume	36,435.5 (14,131.3)	36,838.3 (15,357.9)	0.925
AD signature thickness	2.6 (0.2)	2.7 (0.9)	0.516
BV/CSF index	28.9 (12.9)	29.4 (12.6)	0.884
Hippocampus-to-cortex ratio^b^	0.1 (0.01)	0.1 (0.01)	0.139

Data is presented as mean (standard deviation), except for sex, education, *APOE* ε4 carriers, which correspond to counts and percentages

^a ^Apolipoprotein E (*APOE*) genotype data were available for 64 (37%) individuals (placebo, *n* = 36; donepezil, *n* = 28)

^b ^Hippocampus-to-cortex ratio values were multiplied by 100 to facilitate interpretation

*FCSRT* Free and Cued Selective Reminding Test, *CDR-SOB* clinical dementia rating-sum of boxes, *MMSE* Mini-Mental State Examination, *ADAS-COG-MCI* Alzheimer’s Disease Assessment Scale-cognitive subscale, mild cognitive impairment version, *TMT* Trail Making Test, *AD* Alzheimer’s disease, *BV* brain volume, *CSF* cerebrospinal fluid, *AD signature cortical thickness* entorhinal, inferior temporal, middle temporal, and fusiform gyri thickness

### Characterization of MRI subtypes—continuous subtyping approach

#### Baseline characteristics of MRI subtypes using the continuous subtyping dimensions

**Fig. 3:**
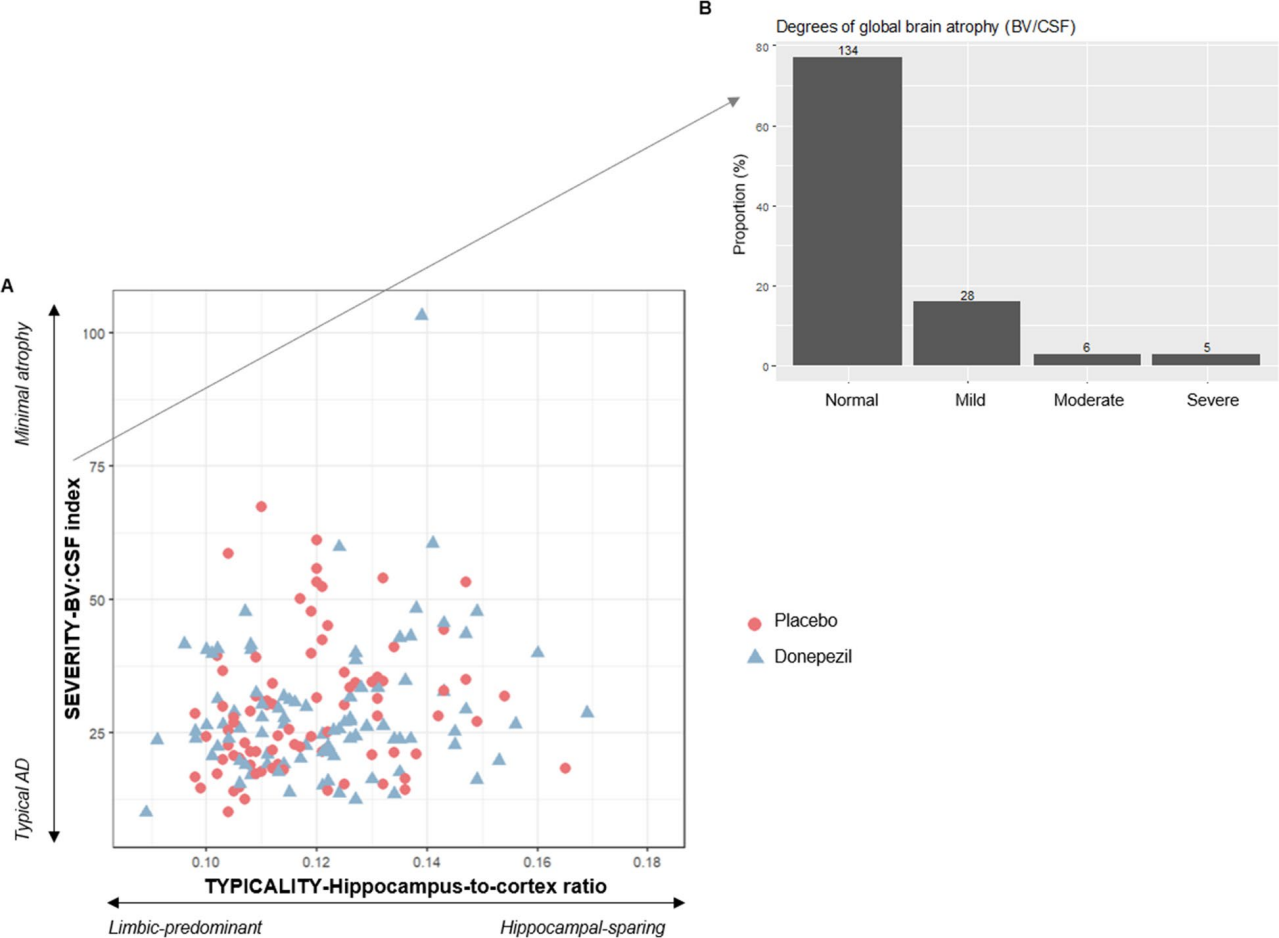
Baseline MRI patterns characterized on continuous scales of subtyping dimensions. **A** Scatterplot of the hippocampus-to-cortex ratio (typicality subtyping dimension) by BV/CSF index (severity subtyping dimension). **B** Classification of MCI individuals according to the degree of global brain atrophy after applying clinical cut-offs on BV/CSF index (severity subtyping dimension). Note: BV = brain volume; CSF = cerebrospinal fluid

We primarily characterized MRI subtypes using continuous scales of the typicality and the severity dimension. Figure 3A displays the distribution of individuals along the severity dimension (i.e. BV/CSF index) and the typicality dimension (i.e. hippocampus-to-cortex ratio). Along the severity dimension, most individuals showed a BV/CSF index towards a minimal atrophy-like MRI pattern. We then classified the individuals according to age-adjusted clinical cut-offs for the BV/CSF index (Fig. 3B). Among participants, 77% were classified as having normal brain atrophy for their age, while only 16% were classified as having mild and 6% as having moderate/severe brain atrophy. Along the typicality dimension, most individuals showed a hippocampus-to-cortex ratio towards the limbic-predominant-like MRI pattern (Fig. 3A).

#### The effect of donepezil treatment: primary MRI efficacy measures

**Fig. 4:**
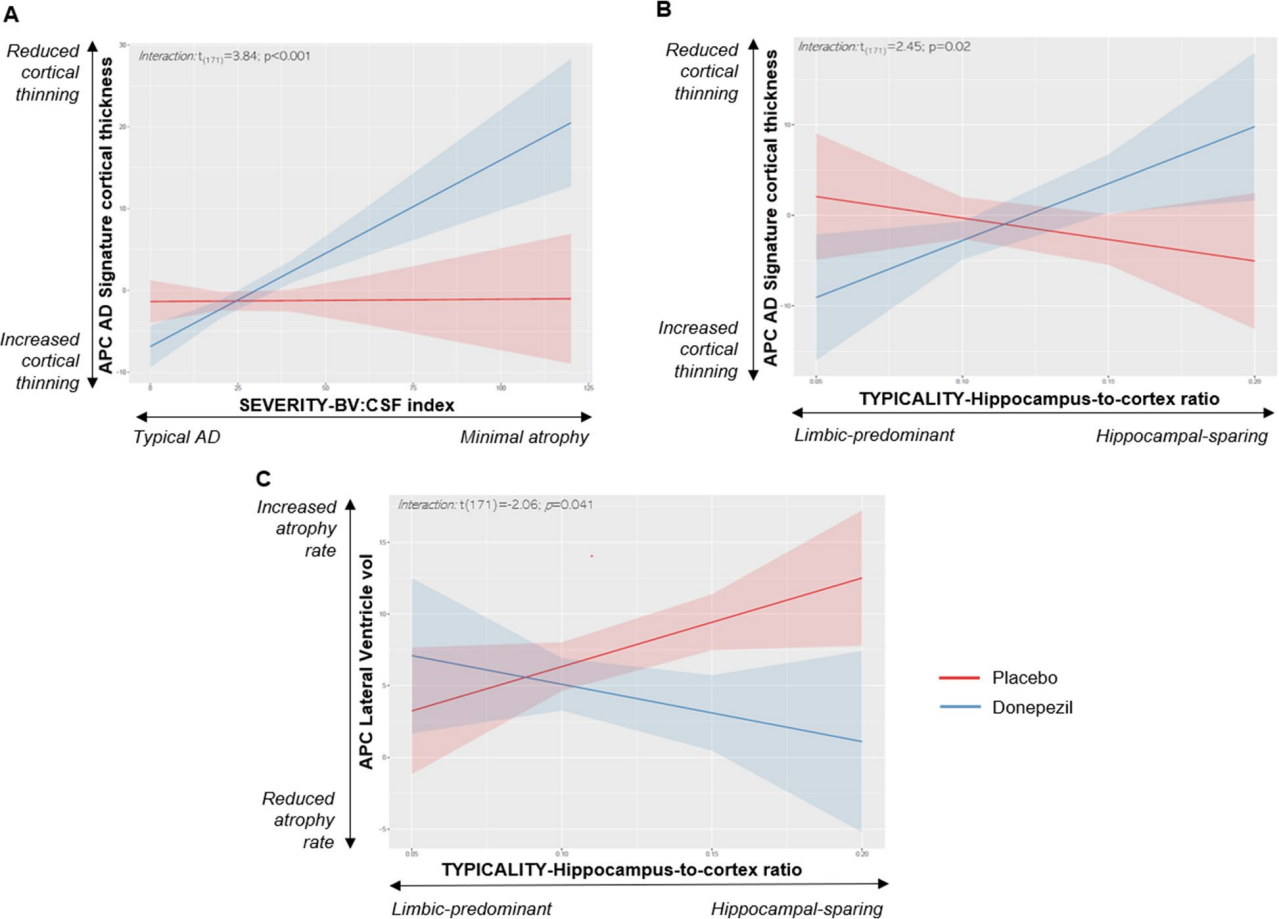
Interaction plots between severity/typicality subtyping dimensions (X axis) and treatment (Y axis) in APC of AD signature cortical thickness which includes entorhinal, inferior temporal, middle temporal, and fusiform gyri thickness (**A**, **B**) and APC of lateral ventricle volume (**C**)

Results from the multiple lineal regression models are summarized in Table 2. Significant interactions of treatment with subtyping dimensions are plotted in Fig. 4.

**Table 2:** Regression analysis testing for interactions between treatment and the subtyping dimensions to predict APC in MRI measures

	***F***	***R***^***2***^	***B***	***p***
***APC hippocampal volume***	*1.1*	*0.04*	*-*	*0.340*
Treatment			− 2.0	0.660
BV/CSF index			0.1	0.810
Hippocampus-to-cortex ratio			− 17.7	0.740
Age			− 0.03	0.710
MRI field strength			0.2	0.820
BV/CSF index by treatment			0.01	0.930
Hippocampus-to-cortex by treatment			25.2	0.490
***APC ventricles volume***	*4.4*	*0.1*	*-*	**< *0.001***
Treatment			8.9	0.138
BV/CSF index			− 0.1	0.710
Hippocampus-to-cortex ratio			61.8	0.042
Age			− 0.04	0.660
MRI field strength			0.4	0.750
BV/CSF index by treatment			− 0.1	0.310
Hippocampus-to-cortex by treatment			− 101.8	**0.041**
***APC total grey matter volume***	*2.1*	*0.1*	*-*	***0.050***
Treatment			2.5	0.450
BV/CSF index			0.04	0.014
Hippocampus-to-cortex ratio			4.6	0.270
Age			− 0.1	0.910
MRI field strength			1.4	0.051
BV/CSF index by treatment			0.1	0.130
Hippocampus-to-cortex by treatment			− 27.7	0.310
***APC AD signature cortical thickness***	*2.5*	*0.1*	***-***	***0.030***
Treatment			− 2.2	0.175
BV/CSF index			0.01	0.808
Hippocampus-to-cortex ratio			− 71.7	0.230
Age			− 0.1	0.340
MRI field strength			1.7	0.100
BV/CSF index by treatment			0.1	**< 0.001**
Hippocampus-to-cortex by treatment			16.4	**0.020**

Values correspond to *R*^*2*^ and its statistical significance for each model. For each predictor and interaction in the models, beta values (*B)* and their *p* values are reported *APC* annual percentage of change, *BV* brain volume, *CSF* cerebrospinal fluid, *AD signature cortical thickness* entorhinal, inferior temporal, middle temporal, and fusiform gyri thickness

We found significant interactions between treatment group and the hippocampus-to-cortex ratio for the AD signature cortical thickness (B = 16.39; *p* = 0.020) and volume of the lateral ventricles (B =  − 101.75; *p* = 0.041). This indicates that donepezil-treated individuals had less cortical thinning and ventricles expansion over time (APCs) than placebo individuals, but particularly if they had a more hippocampal-sparing-like MRI pattern. In contrast, APCs of AD signature cortical thickness and lateral ventricles volume were comparable between donepezil and placebo groups if individuals had a more limbic-predominant-like MRI pattern.

We also found a significant interaction between treatment and the BV/CSF index for the AD signature cortical thickness (B = 0.11; *p* < 0.001). This indicates that donepezil-treated individuals had less cortical thinning over time (APC) than placebo individuals, particularly if they had a more minimal atrophy-like MRI pattern. In contrast, the APCs of AD signature cortical thickness were comparable between donepezil and placebo groups if individuals had a more typical-AD-like MRI pattern.

#### The effect of donepezil treatment: secondary cognitive efficacy measures

The interactions between treatment and MRI subtypes were not significant in the prediction of the percentage of change in any of the cognitive measures (Supplementary Table 2).

### Characterization of MRI subtypes—conventional categorical approach

#### Baseline characteristics of MRI subtypes using the conventional categorical approach

Complementary to the approach based on continuous dimensions of heterogeneity, we also characterized MRI subtypes using the conventional categorical subtyping approach. Supplementary Fig. 1 shows the distribution of the categorical subtype patterns. In line with the results from the continuous approach described in the previous section, the most common patterns were *minimal atrophy* (*n* = 90) and *limbic-predominant* (*n* = 67), which represented 52 and 39% of the individuals, respectively. Only 9% of the individuals were classified as *hippocampal-sparing* (*n* = 9) or *typical AD* (*n* = 7). Baseline characteristics of the subtypes are displayed in Supplementary Table 1.

#### The effect of donepezil treatment

The mixed ANOVAs showed that the interaction between treatment and categorical MRI subtypes did not reach the level for statistical significance in any of the MRI or cognitive efficacy measures (Supplementary Table 3).

## Discussion

The core result of this study was that individuals with MCI show differential responses to donepezil treatment depending on their MRI patterns at baseline. This finding emphasizes the role of biological heterogeneity when it comes to evaluating the response to AChE-Is treatments. Overall, our data showed that MCI individuals with an MRI pattern indicative of minimal atrophy- or a more hippocampal-sparing-like subtype have a reduced rate of atrophy in MRI outcome measures after 1 year of donepezil treatment. Furthermore, as hypothesized, assessing MRI subtypes in the form of continuous severity and typicality dimensions was more sensitive to these differences in the response to donepezil compared to the conventional categorical subtyping approach.

Our findings highlight that the differential response to donepezil was better captured by a continuous characterization of subtyping dimensions than by a conventional categorical subtyping approach. Although most previous studies have used the conventional categorical subtyping approach [18, 19, 21, 22], recent studies have shown the benefits of using continuous dimensions to assess biological heterogeneity in AD [24, 28]. Our results are in line with emerging literature showing that characterizing individuals with AD on continuous dimensions is more sensitive to subtle differences in AD biomarker profiles (atrophy on MRI or tau radioligand uptake on PET). Continuous dimensions allow to better predict longitudinal associations with rates of atrophy, and it better captures regional vulnerabilities to AD and non-AD (co)-pathologies [24, 28]. Using the continuous subtyping approach may also help to better understand the biological heterogeneity in the prodromal stages of the disease. For example, in MCI, brain changes are still mild, and part of this population will never progress to AD, so individuals are usually classified into the minimal atrophy subtype when using a categorical method based on pre-defined cut points developed for AD dementia [45]. On the contrary, continuous dimensions avoid the use of arbitrary cut points and may be more fine-grained to capture variance in the data, still mostly within the range of minimal atrophy. A recent neuropathological study also showed that using severity and typicality as continuous dimensions on ante-mortem MRI captures the presence and regional distribution of different brain pathologies in individuals with AD dementia [28]. This high sensitivity of subtyping dimensions as continuous phenomena opens an opportunity to use this continuous approach in clinical trials to better target candidates for new studies, develop new predictors of short- and long-term response to treatment and, ultimately, reach the goals of precision medicine in neurodegenerative diseases.

In the current cohort, the continuous subtyping approach revealed two predominant MRI patterns. The first pattern was characterized by a low severity and low typicality (i.e. towards a minimal atrophy-like MRI pattern). The second pattern was characterized by high severity but low typicality (i.e. towards a limbic-predominant-like MRI pattern). As a validation, we found the same patterns when we classified the individuals using the conventional categorical approach (52% minimal atrophy and 38% limbic-predominant). These frequency values are consistent with a previous report using visual rating scales to classify MCI individuals from the ADNI cohort into MRI subtypes [45]. The higher frequency of individuals with a minimal atrophy MRI pattern as compared to that observed in individuals with AD was expected because MCI is postulated as a prodromal stage of neurodegenerative disease and, thus, the neuronal damage is still mild [46]. The high frequency of the limbic-predominant MRI pattern contrasts with other studies in MCI and AD, which is generally close to 25% [18, 19, 21, 22]. The high frequency of limbic-predominant individuals in the Hippocampus Study might be explained by the inclusion criteria favouring MCI individuals with an amnestic syndrome characterized by a significant impairment of memory that does not benefit from cueing, which is strongly associated with hippocampal damage [47, 48].

We found that, compared to placebo, MCI individuals showed a slower rate of atrophy in cortical regions that conform the AD cortical thickness signature. A previous study on the same cohort had already shown promising results that indicate a significant impact of donepezil treatment in preserving cortical thickness in individual with MCI [13]. However, statistical significance of those results did not survive multiple comparison corrections. Our current findings confirm the stability of the cortical thickness APC in MCI individuals treated with donepezil, but this effect may be better captured when using composite measures (e.g. AD signature cortical thickness) rather than using a cortical thickness measure from multiple regions (hence reducing multiple comparisons). Further, reducing the heterogeneity through typicality and severity dimensions in our current study could also help capturing significant treatment effects as compared with the previous analysis [13].

Our study also adds evidence suggesting that the effect of donepezil on stabilizing AD signature cortical thickness depends on baseline MRI pattern of atrophy. Using the continuous subtyping approach, we found that those MRI individuals with minimal atrophy- or hippocampal-sparing-like MRI patterns had a slower rate of atrophy in the AD cortical thickness signature. In contrast, MCI individuals with MRI patterns including substantial atrophy in medial temporal structures (towards typical AD or limbic-predominant-like MRI patterns) showed rates of atrophy comparable to the placebo group. A better response to donepezil observed in individuals with minimal atrophy was expected but has not been demonstrated before. This finding is in line with previous reports that encourage to treat patients at early stages of the disease since the neuronal damage at those stages is still mild and treatment may effectively compensate functional deficits [46].

Interestingly, individuals with the hippocampal-sparing-like MRI pattern also showed better response to donepezil in the current study. Firstly, this suggests that severity and typicality dimensions are independent in the prediction of effects to donepezil. Secondly, this finding is consistent with a previous study on individuals with AD undergoing a cholinergic treatment based on encapsulated cell bio-delivery of nerve growth factor (NGF) to the basal forebrain [26]. In that previous study, individuals classified as hippocampal-sparing AD had slower rates of atrophy in the precuneus and hippocampus after NGF treatment. Other data also suggest that cholinergic treatment has a greater effect in individuals that have less atrophy in medial temporal lobe structures [31]. The findings on minimal atrophy and hippocampal-sparing patterns indicate that a relatively intact hippocampus may be the best prognostic factor of a good response to cholinergic treatment in the prodromal stage of AD. This is in line with the cholinergic deficit hypothesis [49] and the overall neuropharmacology of AChE-Is [6]. The hippocampus, the entorhinal cortex, and other medial temporal structures are key regions within the cholinergic system, receiving major cholinergic input from the nucleus basalis of Meynert in the basal forebrain [50]. Therefore, while donepezil could help to increase the acetylcholine availability, a pronounced atrophy in the hippocampus and other medial temporal structures (in limbic-predominant and typical AD subtypes) may limit donepezil efficacy because of a lower number of neurons benefitting from the increased acetylcholine availability. In this sense, individuals with sparing of the hippocampus and other medial temporal structures (minimal atrophy and hippocampal-sparing subtypes) may benefit more from donepezil treatment. The better response to donepezil observed in individuals with hippocampal-sparing-like MRI patterns might be also explained by comorbidities with other pathologies, such as Lewy body-related pathology. Hippocampal-sparing is the most common pattern of atrophy among patients with dementia with Lewy bodies (DLB) [51], and better response to AChE-Is treatment has been observed in patients with DLB compared to AD [52]. In fact, DLB patients with greater hippocampal atrophy have poorer response to AChE-I treatment [53]. Another possible explanation is that the cholinergic system is differentially vulnerable among AD subtypes. A recent study demonstrated that individuals with AD who were classified as hippocampal-sparing according to cortico-limbic patterns of neurofibrillary tangles at autopsy, had a more pronounced degeneration of the nucleus basalis of Meynert, one of the main nuclei of the cholinergic system with important cortical projections [54]. Based on the relevance of the nucleus basalis if Meynert for targeted treatment by AChE-Is, the greater involvement of the cholinergic system in the hippocampal-sparing subtype might explain its higher rate of response to donepezil in this study. Further research including in vivo measures of cholinergic nuclei degeneration is warranted to better understand the differential responses to AChE-Is across biological subtypes. Altogether, these findings may serve as a preliminary pharmacological support to the “distinct cortical” hypothesis of the hippocampal-sparing subtype [55]. That hypothesis postulates that the hippocampal-sparing pattern develops from the minimal atrophy pattern through a distinct pathway of spread of pathology and neurodegeneration, deviating from the canonical pathway of spread postulated by the Braak’s staging system [56]. That distinct pathological pathway may explain a distinct response to AChE-I treatment.

Consistently with a previous study in this cohort [12], there was a non-significant effect of donepezil treatment on cognitive measures along the MRI subtyping dimensions or across the MRI categorical subtypes. As it has been previously argued, cognitive tests might be less sensitive than biological data in detecting significant changes in brain functioning at early stages of MCI. Furthermore, the duration of this clinical trial is 12 months. At the MCI stage, cognitive decline is mild, and normally progress slowly over time. We hypothesize that our cognitive tests are not yet capturing these slow and mild changes and thus any potential effects of treatment.

This study has some limitations. The sample size is relatively small, which could compromise statistical power. This limitation mostly affected statistical testing when using the categorical subtyping approach. The classification of individuals into four categorical smaller groups increases the number of comparisons and reduces statistical power. However, we demonstrated that the characterization of subtypes using continuous dimensions can overcome this limitation, which can be an important step forward in the implementation of the current framework for biological subtypes in clinical trials [17]. Considering that the recruitment of large cohorts is generally limited in clinical trials of AD or MCI, the continuous subtyping approach emerges as a preferred alternative. Larger cohorts would help to further investigate the potential modulation of other key factors, such as age, sex, or disease duration, on donepezil response along MRI subtyping dimensions or across MRI categorical subtypes. Furthermore, the current clinical trial did not include information on biomarkers of amyloid-beta and tau pathology, which might be useful to assess the effects of donepezil on AD neuropathology in vivo. Additional data on these AD biomarkers together with other clinical features and psychiatric conditions might also be helpful to better characterize the MCI patients and understand the reasons behind a better response to donepezil in the patients with an MRI pattern towards minimal atrophy and hippocampal-sparing subtypes. Although our main objective was to characterize biological heterogeneity in atrophy patterns, the ability of MRI to detect overt atrophy may be limited at the MCI stage.

## Conclusion

In conclusion, we demonstrated that the response to donepezil treatment in MCI individuals differs depending on MRI subtypes. Our data suggest that donepezil should be better prescribed for MCI individuals with minimal atrophy- or hippocampal-sparing-like MRI patterns. Future studies will need to expand on these findings using long-term follow-up assessments and biomarker-based protocols. Such future studies may help to clarify whether these specific subtypes exhibit different AD biomarker trajectories and significantly delayed cognitive and functional decline under long-lasting donepezil treatment. Our data also confirmed that operationalizing biological heterogeneity as a continuous phenomenon has higher sensitivity in capturing differences in the response to donepezil over the conventional categorical subtyping approach, particularly in individuals at prodromal stages of the disease and in cohorts limited in size. This finding adds to the emerging notion that continuous subtyping is superior to categorical subtyping [24, 28]. Finally, this study illustrates that considering the underlying biology is important for optimizing more personalized treatment approaches. This is a step towards precision medicine in neurodegenerative disorders such as AD [16].

### Supplementary Information

**Additional file 1: Supplementary Figure 1.** Distribution of MCI subtypes based on patterns of brain atrophy from visual rating scales. **Supplementary Table 1.** Characteristics of MCI subtypes using the conventional categorical subtyping approach. **Supplementary Table 2.** Regression analysis testing for interactions of the Subtyping Dimensions (continuous subtyping approach) by Treatment to predict percentage of change in cognitive measures. **Supplementary Table 3.** Mixed ANCOVA-interactions effects of the MCI subtypes (conventional categorical subtyping approach) by Treatment on the percentage of change of MRI and cognitive measures.

## Data Availability

The datasets used and/or analysed during the current study are available from the corresponding author on reasonable request.

## Abbreviations

AD: Alzheimer’s disease
ADAS-COG-MCI: Alzheimer’s Disease Assessment Scale-cognitive subscale
ADNI: Alzheimer’s disease Neuroimaging Initiative
ANOVA: Analysis of variance
APC: Annual percentage of change
APOE: Apolipoprotein E
BV: Brain volume
CDR-SOB: Clinical dementia rating-sum of boxes
CSF: Cerebrospinal fluid
DLB: Dementia with Lewy bodies
FCSRT: Free and Cued Selective Reminding Test
GCA-F: Global cortical atrophy-frontal
MCI: Mild cognitive impairment
MMSE: Mini-Mental State Examination
MRI: Magnetic resonance imaging
MTA: Medial temporal atrophy
PA: Posterior atrophy
TMT: Trail making test
