# Correction: Differential response to donepezil in MRI subtypes of mild cognitive impairment

**DOI:** 10.1186/s13195-023-01320-8

**Published:** 2023-10-14

**Authors:** Patricia Diaz‑Galvan, Giulia Lorenzon, Rosaleena Mohanty, Gustav Mårtensson, Enrica Cavedo, Simone Lista, Andrea Vergallo, Kejal Kantarci, Harald Hampel, Bruno Dubois, Michel J. Grothe, Daniel Ferreira, Eric Westman

**Affiliations:** 1 Department of Radiology, Mayo Clinic, Rochester, MN USA; 2 Division of Clinical Geriatrics, Center for Alzheimer Research, Department of Neurobiology, Care Sciences and Society, Karolinska Institutet, Huddinge, Sweden; 3 Alzheimer Precision Medicine (APM), Sorbonne University, AP-HP, Pitié-Salpêtrière Hospital, Boulevard de L’hôpital, Paris, France; 4 Unidad de Trastornos del Movimiento, Servicio de Neurología y Neurofisiología Clínica, Instituto de Biomedicina de Sevilla, Hospital Universitario Virgen del Rocío, CSIC, Seville, Spain; 5 Wallenberg Center for Molecular and Translational Medicine, Department of Psychiatry and Neurochemistry, University of Gothenburg, Gothenburg, Sweden; 6 Department of Neuroimaging, Centre for Neuroimaging Sciences, Institute of Psychiatry, Psychology, and Neuroscience, King’s College London, London, UK

**Correction: Alz Res Ther 15, 117 (2023)**

**https://doi.org/10.1186/s13195-023-01253-2**

Following publication of the original article [1], the authors identified an error to the titles and labels of axes in Figs. 3 and 4, as well as the captions of treatment groups on Fig. 4. This error does not affect the accuracy and clarity of the research presented, neither does affect the interpretation of the results. The corrected figures are given below.

**Error:**

- In Fig. 3A (Scatterplot of the hippocampus-to-cortex ratio by BV/CSF index)***,*** labels indicating the MRI subtypes are in the reverse order.
  - In the X axis, which corresponds to the “TYPICALITY-Hippocampus-to-cortex ratio”, labels should go **from “Limbic-predominant” (on the left) to “Hippocampal-sparing” (on the right)**.
  - In the Y axis, which corresponds to the “SEVERITY-BV:CSF index”, labels should go from **“Minimal atrophy” (at the top) to “Typical AD” (at the bottom)**.
- In Fig. 4 (interaction plots between severity/typicality subtyping dimension and treatment in the MRI efficacy measures), captions of the lines indicating the treatment groups are in reversed order. **Red lines correspond to the placebo group** (not to the donepezil group), and **blue lines correspond to the donepezil group** (not to the placebo group).
- In Fig. 4C (interaction plot between typicality subtyping dimension and APC of lateral ventricle volume), axes titles are incorrect.
  - X axis title should be “TYPICALITY-Hippocampus-to-cortex ratio”, instead of “SEVERITY-BV:CSF index”.
  - Y axis title should be “APC Lateral Ventricle vol”, instead of "APC AD Signature cortical thickness”. Labels on the Y axis are also incorrect. They should be changed to “Increased atrophy rate” (at the top) and “Reduced atrophy rate” (at the bottom).

**Fig. 3:**
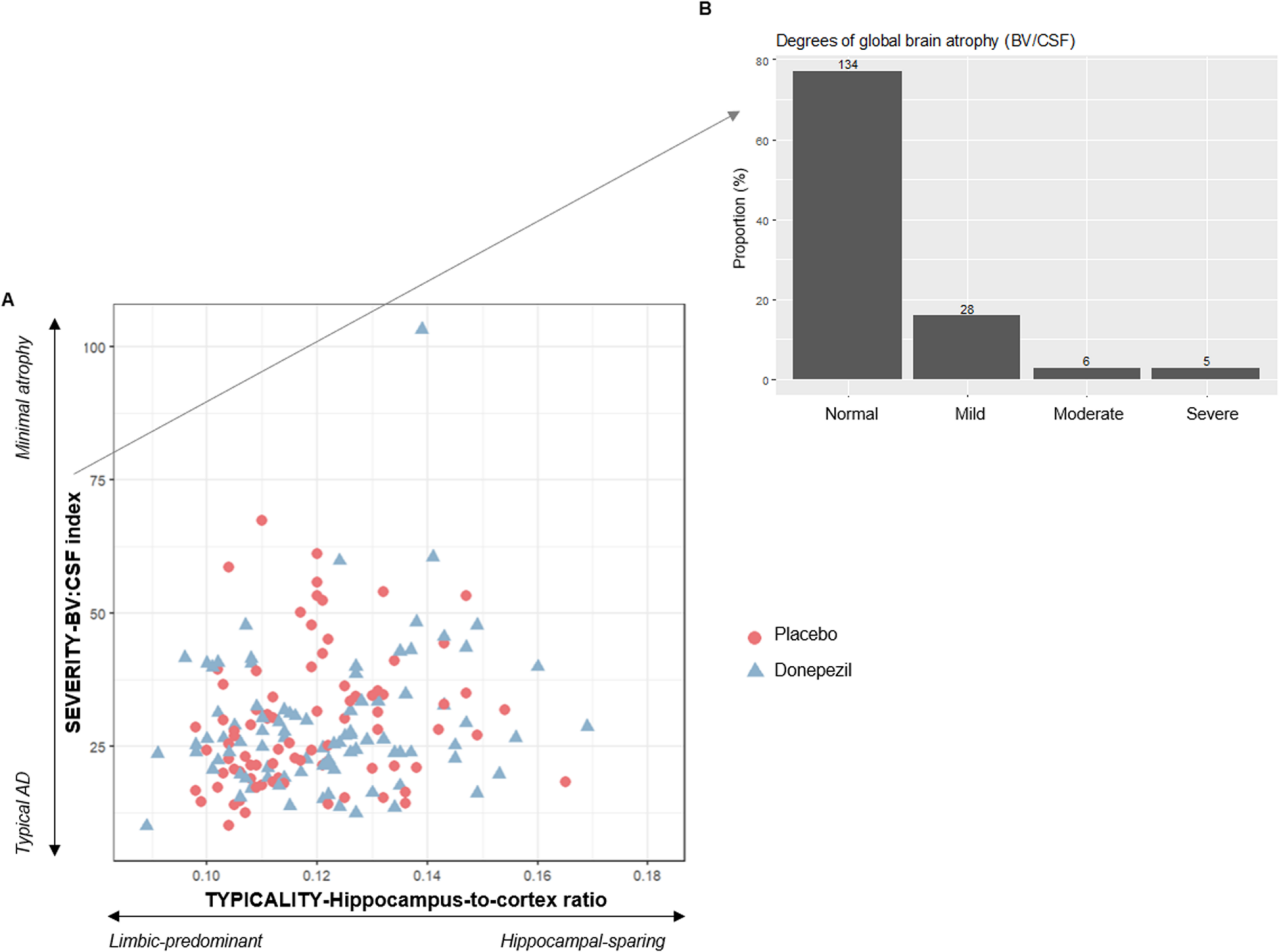
Baseline MRI patterns characterized on continuous scales of subtyping dimensions. **A** Scatterplot of the hippocampus-to-cortex ratio (typicality subtyping dimension) by BV/CSF index (severity subtyping dimension). **B** Classification of MCI individuals according to the degree of global brain atrophy after applying clinical cut-offs on BV/CSF index (severity subtyping dimension). Note: BV = brain volume; CSF = cerebrospinal fluid

**Fig. 4:**
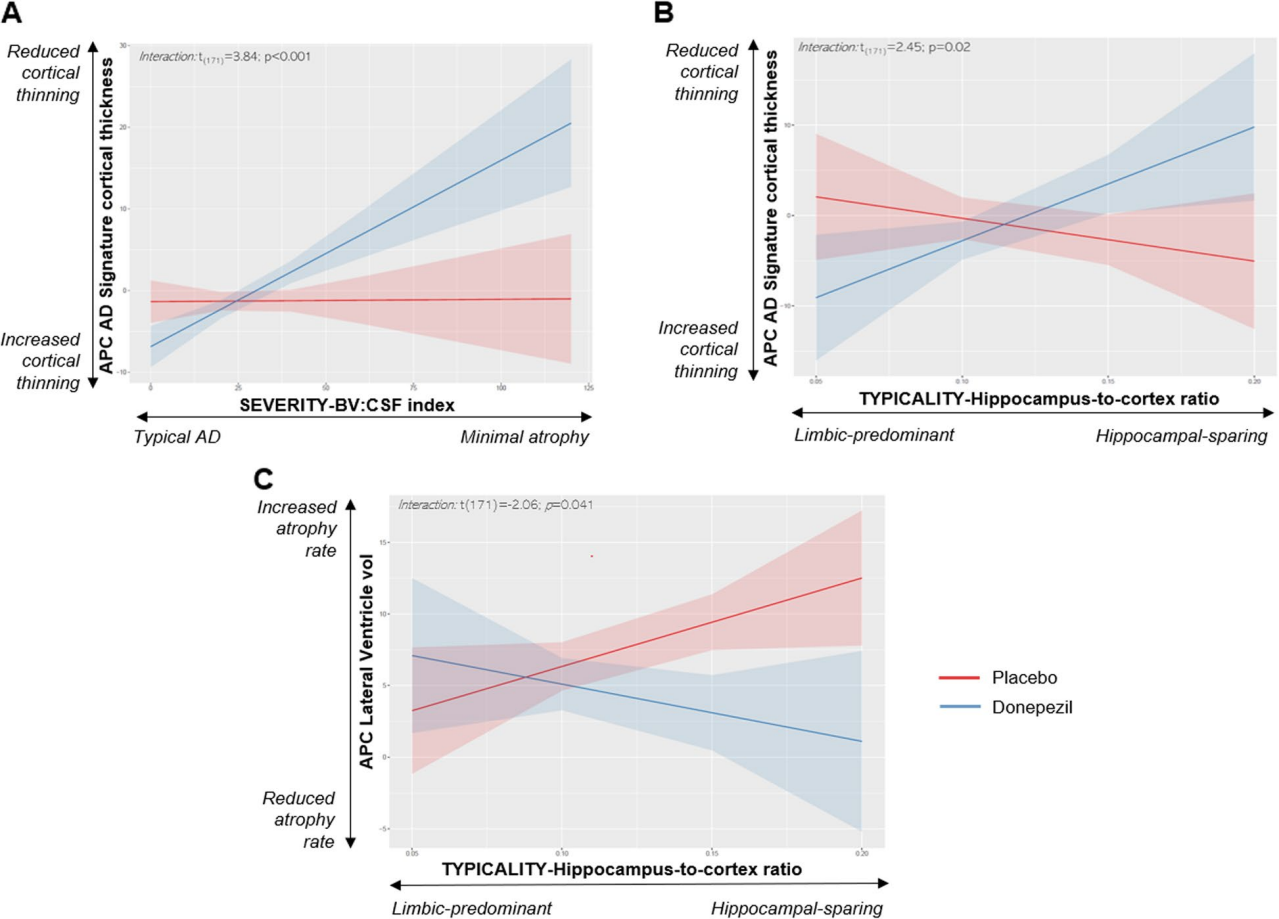
Interaction plots between severity/typicality subtyping dimensions (X axis) and treatment (Y axis) in APC of AD signature cortical thickness which includes entorhinal, inferior temporal, middle temporal, and fusiform gyri thickness **(A, B)** and APC of lateral ventricle volume **(C)**

The original article [1] has been updated.
