# Supplementary material for: Differential response to donepezil in MRI subtypes of mild cognitive impairment
Source: Alzheimers Res Ther. 2023 Jun 23;15:117. doi: 10.1186/s13195-023-01253-2 (PMC10288762; doi:10.1186/s13195-023-01253-2)
Supplement: Supplementary file 1 — Additional file 1: Supplementary Figure 1. Distribution of MCI subtypes based on patterns of brain atrophy from visual rating scales. Supplementary Table 1. Characteristics of MCI subtypes using the conventional categorical subtyping approach. Supplementary Table 2. Regression analysis testing for interactions of the Subtyping Dimensions (continuous subtyping approach) by Treatment to predict percentage of change in cognitive measures. Supplementary Table 3. Mixed ANCOVA-interactions effects of the MCI subtypes (conventional categorical subtyping approach) by Treatment on the percentage of change of MRI and cognitive measures. [file 13195_2023_1253_MOESM1_ESM.docx]

**Supplementary Material**

**
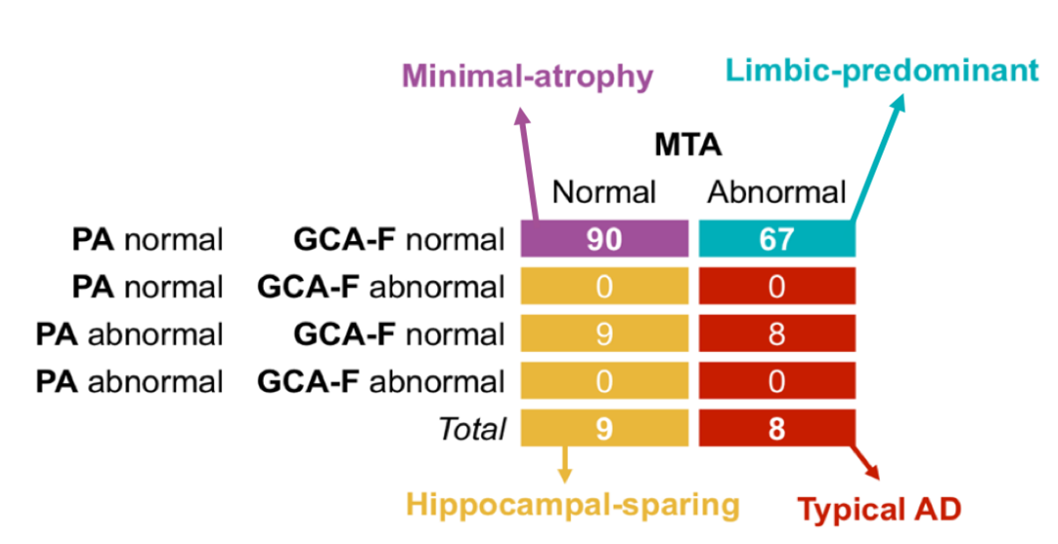
**

**Supplementary Figure 1.** Distribution of MCI subtypes based on patterns of brain atrophy from visual rating scales.

Atrophy in medial temporal, frontal, and posterior regions was measured with the estimation of MTA, GCA-P, and PA visual rating scales, respectively. Scores from each visual rating scales were estimated using the deep learning-based AVRA toolbox. In the three visual rating scales, a score of 0 denotes normal atrophy, whereas scores from 1 to 3 (GFA-F and PA) or 4 (MTA) denote an increasing degree of atrophy. We combined normal and abnormal scores on the visual rating scales to assign participants into one of four categorical subtypes: typical AD, limbic predominant, hippocampal-sparing, or minimal atrophy. The figure shows the distribution of MCI patients who were classified into the categorical subtypes based on their pattern of brain atrophy from the estimated visual rating scales.
Note: AD=Alzheimer’s disease; GCA-F=general cortical atrophy – frontal subscale; MTA=medial temporal atrophy scale; PA=posterior atrophy scale.

| **Supplementary Table 1** Characteristics of MCI subtypes using the categorical subtyping method | | | | | |
| --- | --- | --- | --- | --- | --- |
|  | **Typical AD**  **(n=7)** | **Limbic-**  **predominant**  **(n=67)** | **Hippocampal**  **sparing**  **(n=9)** | **Minimal**  **atrophy**  **(n=90)** | ***p*** |
| Treatment (Donepezil/Placebo) | 4/3 | 31/36 | 4/5 | 44/46 | 0.943 |
| Age | 74.6 (4.7) | 75.6 (5.7) | 76 (6.4) | 72.3 (7.1) | **0.015** |
| Sex, female n(%) | 2 (29) | 34 (51) | 5 (56) | 48 (54) | 0.625 |
| Education, n(%) | | | | | 0.975 |
| *No education* | 0 (0) | 0 (0) | 0 (0) | 1 (0) |  |
| *Primary* | 1 (14) | 4 (6) | 0 (0) | 8 (1) |  |
| *Certification of primary* | 2 (29) | 28 (42) | 5 (56) | 38 (43) |  |
| *Secondary* | 1 (14) | 14 (21) | 2 (22) | 19 (21) |  |
| *Higher education* | 3 (43) | 21 (31) | 2 (22) | 23 (26) |  |
| Duration of memory disorders | 51.4 (35.1) | 33.7 (24.9) | 40.2 (26.3) | 34.7 (27.6) | 0.375 |
| Hamilton Depression Rating Scale | 3 (2.3) | 3.4 (2.9) | 2 (1.5) | 2.9 (2.8) | 0.116 |
| *APOE* ε4 carriers, n (%)^a^ | 2 (50) | 10 (45) | 2 (40) | 18 (55) | 0.884 |
| Follow-up MRI (months) | 12 (0.1) | 11.5 (1.6) | 11.4 (2) | 11.7 (1.3) | 0.887 |
| CDR-SOB | 1.5 (0.7) | 1.8 (1.0) | 1.2 (0.7) | 1.5 (0.9) | 0.144 |
| MMSE | 26.3 (1.7) | 26.2 (2.4) | 25.6 (2.2) | 26 (2.5) | 0.873 |
| ADAS-COG-MCI | 13.5 (4.3) | 12.8 (4.6) | 12 (4.5) | 11.4 (4) | 0.232 |
| TMT-A (Time) | 61.6 (31.2) | 62.5 (25.1) | 67.6 (30) | 62 (32) | 0.743 |
| TMT-B (Time) | 167.9 (56.2) | 149.2 (60.7) | 150.6 (69.6) | 150.1 (63.8) | 0.845 |
| Benton test | 6.9 (2.1) | 6.9 (2) | 6.4 (2) | 7.2 (1.9) | 0.444 |
| Isaacs test (15 items) | 23.8 (5.7) | 23.6 (4.4) | 26.9 (7) | 25.7 (5.4) | **0.039** |
| Isaacs test (60 items) | 45 (5.5) | 47.7 (9) | 55.8 (17.2) | 51.4 (13) | **0.042** |
| Field strength 3T, n (%) | 1 (14) | 13 (19) | 1 (11) | 30 (34) | 0.123 |
| Total hippocampal volume | 5553.3 (568.9) | 5809.6 (641.6) | 6276.4 (582.7) | 6672.9 (835.1) | **<0.001** |
| Total gray matter volume | 542219.1 (18721.8) | 535037.9 (27627.9) | 556774.7 (36866.8) | 565515.8 (27164.8) | **<0.001** |
| Lateral ventricular volume | 47586.8 (11495.4) | 45500.2 (15651.8) | 35997.9 (3814.4) | 29235.3 (10059.4) | **0.001** |
| AD signature thickness | 2.7 (0.2) | 2.6 (0.2) | 2.7 (0.2) | 2.8 (0.2) | **<0.001** |
| BV/CSF index | 21.1 (5.4) | 22.2 (7.6) | 26.3 (3.3) | 35.4 (13.7) | **<0.001** |
| Hippocampus-to-cortex ratio^b^ | 0.2 (0.1) | 0.2 (0.1) | 0.2 (0.1) | 0.2 (0.1) | **0.001** |
| Data is presented as mean (standard deviation), except for sex, education, APOE ε4 carriers, which correspond to counts and percentages. ^a^Apolipoprotein E (APOE) genotype data were available for 64 (37%) individuals (placebo, n=36; donepezil, n=28). ^b^Hippocampus-to-cortex-ratio values were multiplied by 100 to facilitate interpretation.  FCSRT= Free and Cued Selective Reminding Test; CDR-SOB= clinical dementia rating-sum of boxes; MMSE= Mini Mental State Examination; ADAS-COG-MCI= Alzheimer’s Disease Assessment Scale-cognitive subscale, mild cognitive impairment version; TMT= Trail Making Test; AD= Alzheimer’s disease; BV=brain volume; CSF= cerebrospinal fluid; AD signature cortical thickness = entorhinal, inferior temporal, middle temporal, and fusiform gyri thickness. | | | | | |

| **Supplementary Table 2** Regression analysis testing for interactions of the Subtyping Dimensions (continuous subtyping approach) by Treatment to predict percentage of change in cognitive measures | | | |
| --- | --- | --- | --- |
|  | *F* | *R^2^* | *p* |
| *PC ADAS-COG-MCI* | 0.3 | 0.01 | 0.95 |
| *PC MMSE* | 0.9 | 0.03 | 0.53 |
| *PC TMT-Part A* | 0.6 | 0.02 | 0.70 |
| *PC TMT-Part B* | 0.7 | 0.03 | 0.63 |
| *PC Isaac’s verbal fluency test (15 items)* | 2.0 | 0.04 | 0.43 |
| *PC Isaac’s verbal fluency test (15 items)* | 1.8 | 0.10 | 0.10 |
| *PC Benton test* | 1.1 | 0.04 | 0.35 |
| Values correspond to *R^2^* and its statistical significance for each model. PC=percentage of change; ADAS-COG-MCI= Alzheimer’s Disease Assessment Scale-cognitive subscale, mild cognitive impairment version; TMT= Trail Making Test. | | | |

| **Supplementary Table 3** Mixed ANCOVA-interactions effects of MRI Categorical Subtypes by Treatment on the percentage of change of MRI and cognitive measures. | | |
| --- | --- | --- |
| 1. ***Annual percentage of change of MRI measures*** | | |
|  | ***F_(3,160)_*** | ***p*** |
| *APC Hippocampal volume* | 0.8 | 0.495 |
| *APC Lateral ventricles volume* | 0.9 | 0.467 |
| *APC Gray matter volume* | 0.3 | 0.841 |
| *APC AD signature cortical thickness* | 0.4 | 0.788 |
| 1. ***Percentage of change of cognitive measures*** | | |
|  | ***F_(3,160)_*** | ***p*** |
| *PC ADAS-COG-MCI* | 0.4 | 0.726 |
| *PC MMSE* | 0.6 | 0.639 |
| *PC TMT-Part A* | 0.7 | 0.551 |
| *PC TMT-Part B* | 1.8 | 0.155 |
| *PC Isaac’s verbal fluency test (15 items)* | 2.3 | 0.077 |
| *PC Isaac’s verbal fluency test (15 items)* | 1.2 | 0.296 |
| *PC Benton test* | 0.9 | 0.441 |
| ADAS-COG-MCI= Alzheimer’s Disease Assessment Scale-cognitive subscale, mild cognitive impairment version; APC=annual percentage of change; AD signature cortical thickness= entorhinal, inferior temporal, middle temporal, and fusiform gyri thickness; BV=brain volume; CSF= cerebrospinal fluid; TMT= Trail Making Test | | |
